# Consideration of Cybersecurity Risks in the Benefit-Risk Analysis of Medical Devices: Scoping Review

**DOI:** 10.2196/65528

**Published:** 2024-12-24

**Authors:** Oscar Freyer, Fatemeh Jahed, Max Ostermann, Christian Rosenzweig, Pascal Werner, Stephen Gilbert

**Affiliations:** 1 Else Kröner Fresenius Center for Digital Health Dresden University of Technology Dresden Germany; 2 Johner Institute Konstanz Germany; 3 Regulatory.me Mebane, NC United States

**Keywords:** cybersecurity, connected medical devices, benefit-risk analysis, risk management, patient safety, regulation, vulnerability assessment

## Abstract

**Background:**

The integration of connected medical devices (MDs) into health care brings benefits but also introduces new, often challenging-to-assess risks related to cybersecurity, which have the potential to harm patients. Current regulations in the European Union and the United States mandate the consideration of these risks in the benefit-risk analysis (BRA) required for MD approval. This important step in the approval process weighs all the defined benefits of a device with its anticipated risks to ensure that the product provides a positive argument for use. However, there is limited guidance on how cybersecurity risks should be systematically evaluated and incorporated into the BRA.

**Objective:**

This scoping review aimed to identify current legal frameworks, guidelines, and standards in the United States, Canada, South Korea, Singapore, Australia, the United Kingdom, and the European Union on how cybersecurity risks should be considered in the BRA of MDs.

**Methods:**

This scoping review followed the PRISMA-ScR (Preferred Reporting Items for Systematic Reviews and Meta-Analyses extension for Scoping Reviews) framework. A systematic literature search of 10 databases was conducted in two phases on July 3, 2024 and September 30, 2024, including the guidance databases of the Food and Drug Administration, the Medical Device Coordination Group, and other International Medical Device Regulators Forum members; the International Medical Device Regulators Forum database; PubMed; and Scopus. Search terms included “cybersecurity,” “security,” “benefit/risk,” “benefit-risk,” and “risk-benefit.” Additional references were identified via citation searching and expert interviews. Inclusion criteria were met if a document was a guideline or standard in force that provided guidance on the BRA or cybersecurity risks of MDs. Documents were excluded when they were not relevant to MDs, they were limited to a subclass of devices, they were about in vitro diagnostic MDs or investigational devices, and the content of the source was insufficient to undertake a scientific analysis. Data were extracted and analyzed using MAXQDA 2022, and the findings were narratively summarized and visualized in figures and tables.

**Results:**

The search identified 150 documents, with 34 (22.7%) meeting the inclusion criteria. These 34 documents included 4 (12%) regulations, 5 (15%) standards, 6 (18%) technical reports, and 19 (56%) guidance documents. While cybersecurity risks were acknowledged in most documents, detailed methods for their integration into the BRA were lacking. Some standards and guidelines provided examples of how to consider cybersecurity risks in the BRA, but a comprehensive and standardized approach was lacking.

**Conclusions:**

This review highlights a substantial gap between the recognition of cybersecurity risks in MDs and the guidance on their incorporation into the BRA. Standardized frameworks are needed to provide clear methods for evaluating cybersecurity risks and their impact on the safety and security of MDs.

## Introduction

### Background

Connected medical devices (cMDs) have become an integral part of modern health care and play a crucial role in diagnosing, monitoring, and treating a wide range of medical conditions [1]. These devices have integrated software or are entirely software based, allowing them to connect to other devices or networks to exchange, transfer, or receive commands and data [1]. Examples include smartwatches, implanted devices often referred to as the Internet of Medical Things [2], and stationary devices such as computed tomography scanners [1,3]. While their connectivity offers multiple advantages, such as real-time disease and physiology monitoring [1,4,5] along with the potential for remote device management and over-the-air updating [6], it also introduces new risks for patients, particularly related to cybersecurity [4,5,7,8]. These risks are not just theoretical but could harm patients [9,10]. “Cybersecurity risks” is used as an umbrella term for risks specifically arising from cybersecurity vulnerabilities and measures that could affect the security (that is, a state in which information assets are protected) and safety (the absence of unacceptable risks to the patient’s health) of a medical device (MD) [11,12]. However, those 2 areas overlap to some degree as security risks could exist that affect the MD’s safety [13]. Thus, cybersecurity risks can lead to harm “to people, property, and the environment” [12].

The relevance of cybersecurity risks is underscored by a rising number of cyberattacks on health care infrastructure [9,14] while, at the same time, the US Food and Drug Administration (FDA) reports a substantial number of adverse events related to cybersecurity vulnerabilities [15] and has even recalled several devices because of them [16,17]. Regulatory frameworks such as the Medical Device Regulation (EU_MDR) in the European Union (EU) and the Federal Food, Drug, and Cosmetic Act (FD&C Act) in the United States mandate that manufacturers secure cMDs against these vulnerabilities to ensure that the risks for patients are as minimal as possible to guarantee a high level of health and safety protection [18-20]. Those high-level requirements are often further specified in guidance documents provided by authorities such as the Medical Device Coordination Group (MDCG) for the EU, the FDA for the United States, or the Therapeutic Goods Administration (TGA) for Australia [13,21,22].

To assess whether these requirements for health and safety protection are met, manufacturers are often obligated under legislation in multiple regions, including the United States, Australia, and the EU, to carry out a benefit-risk analysis (BRA), which determines whether the benefits of an MD outweigh its risks [18-20,23]. This important step in the approval process weighs all defined benefits of a device with its anticipated risks (including the risk detected for similar devices) considering the device’s intended use to ensure that the product as a whole provides a positive argument for use [24].

As guidance documents often prescribe a BRA but rarely prescribe a precise methodology, there are multiple qualitative, quantitative, and semiquantitative methods in use [25,26], such as the multicriteria decision analysis (MCDA) [27], the quantitative BRA [28], or the FDA’s Benefit-Risk Framework (BRF) [24]. Despite the prevalence of qualitative methods among manufacturers [29], there is currently no established standard for their use. Qualitative methods are often critiqued for their subjectivity and lack of rigor, whereas quantitative methods are often regarded as superior by multiple researchers [28,30,31]. However, the applicability of quantitative methods, which often originate from the pharmaceutical industry, is limited for MDs due to the inherent challenges in quantifying risks associated with aspects of their use (eg, cybersecurity risks) [25]. The importance of the consideration of cybersecurity risks in the BRA is addressed in several guidance documents [13,22,32]. However, the guidance fails to provide clear instructions on how to evaluate and incorporate these often challenging-to-assess risks into the BRA [11,12,25].

### Objectives

To address the existing ambiguities regarding the BRA and cybersecurity, this review aimed to identify the current legal frameworks, guidelines, and standards in the United States, Canada, South Korea, Singapore, Australia, the United Kingdom, and the EU on how cybersecurity risks should be considered in the BRA of MDs.

## Methods

This scoping review was conducted according to the PRISMA-ScR (Preferred Reporting Items for Systematic Reviews and Meta-Analyses extension for Scoping Reviews) guidelines [33]. The completed PRISMA-ScR checklist can be found in Table S1 in Multimedia Appendix 1 [33].

### Search Strategy

The first literature search was conducted on July 3, 2024, and a second search was conducted on September 30, 2024, in the regulatory guidance databases of 7 International Medical Device Regulators Forum (IMDRF) member states that provide relevant guidance in English: the US FDA guidance database, the EU MDCG document database, the Australian TGA guidance database, the Health Canada guidance document database, the South Korean Ministry of Food and Drug Safety (MFDS) regulation database, the UK guidance and regulation database (with a filter for Medicines and Healthcare Products Regulatory Agency [MHRA] documents), and the Singaporean Health Sciences Authority (HSA) database of guidance documents for MDs. In addition, the IMDRF guidance document database, Scopus, and PubMed were searched. Each guidance database was searched using the terms “cybersecurity,” “security,” “benefit-risk,” “benefit/risk,” and “risk-benefit.” Scopus was searched using the following search string: TITLE-ABS-KEY ( ( cybersecurity OR “information security” OR “cyber security” ) AND ( “medical device*” OR “health device*” ) AND ( “benefit-risk” OR “risk-benefit” OR “benefit/risk” )). PubMed was searched using the following search string: (cybersecurity OR “information security” OR “cyber security”) AND (“medical device*” OR “health device*”) AND (“benefit-risk” OR “risk-benefit” OR “benefit/risk”). In addition, a reference search of the included guidelines was conducted to identify any other relevant standards or guidelines referenced in the official guidance documents, and 2 experts on the regulation of cybersecurity in MDs (authors CR and PW) were asked to provide the most recent guidance documents that are not yet referenced in the official guidelines. The data from all the identified guidelines were exported and gathered in an electronic database (Microsoft Excel for Mac; version 16.86.3).

### Inclusion and Exclusion Criteria

Sources were included in this review if they met the following inclusion criteria: (1) they were a regulation, guideline, or standard or an academic paper describing regulations, guidelines, or standards; (2) they were in force (applicable for regulations, guidelines, and standards); and (3) they delivered guidance for the BRA of MDs or cybersecurity risks of MDs. Sources were excluded when (1) they were not relevant to MDs, (2) they were limited to a specific subclass of MDs and not generalizable (eg, digital diabetes devices), (3) they were about in vitro diagnostic MDs or investigational devices, and (4) the content of the source was insufficient to undertake a scientific analysis.

### Study Selection

The titles and summaries of the identified sources were screened by 2 independent researchers between July 3, 2024, and October 6, 2024, to evaluate whether they met the criteria for inclusion. In case of disagreements, a third independent reviewer was consulted. After the initial screening, 1 reviewer screened the full text of the included guidelines for eligibility.

### Data Extraction and Analysis

Data extraction and analysis were conducted using MAXQDA 2022 (VERBI GmbH). One researcher identified keywords related to the BRA and cybersecurity risks within each document. Thereafter, any guidance and recommendations related to both topics and to the intersection of both were extracted. The extracted data and the relationships between different data sources were then synthesized using figures, tables, and narrative summaries. Recommendations regarding cybersecurity risks in the BRA provided in the documents were listed.

## Results

### Search Results

**Figure 1:**
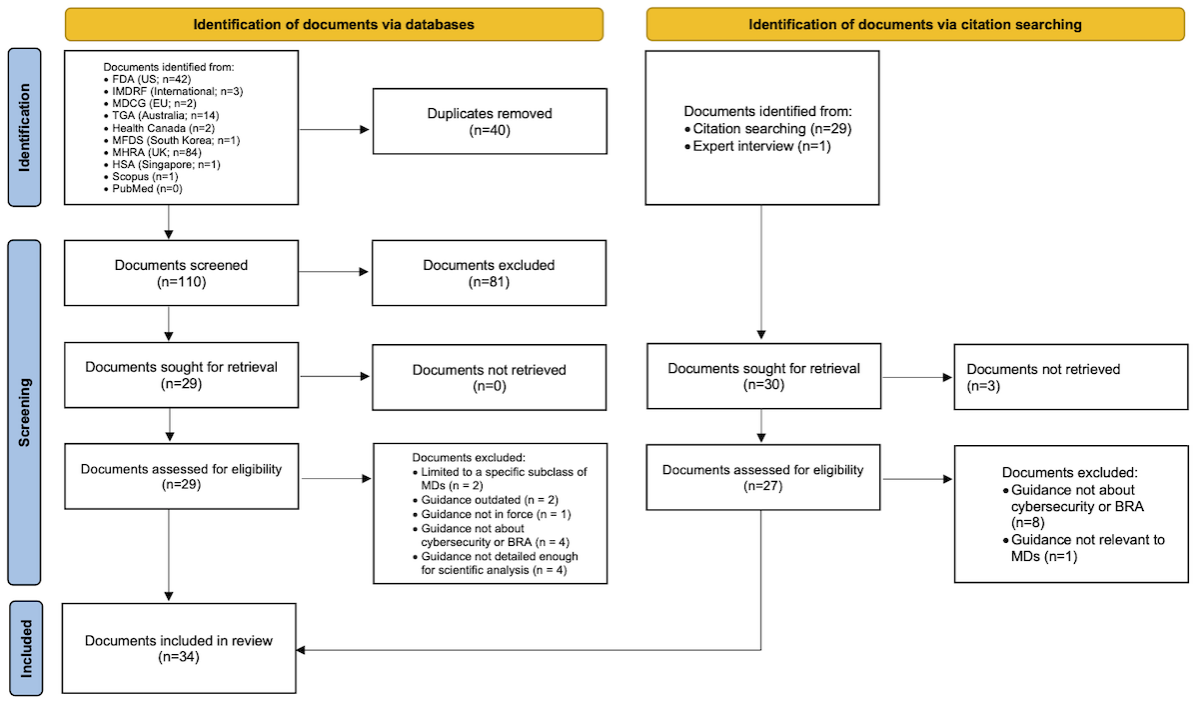
Flowchart of the screening process. This flowchart follows the template provided by Page et al [34]. BRA: benefit-risk analysis; EU: European Union; FDA: Food and Drug Administration; HSA: Health Sciences Authority; IMDRF: International Medical Device Regulators Forum; MD: medical device; MDCG: Medical Device Coordination Group; MFDS: Ministry of Food and Drug Safety; MHRA: Medicines and Healthcare Products Regulatory Agency; TGA: Therapeutic Goods Administration.

The systematic search of the 10 databases resulted in 150 documents. Of those 150 documents, 16 (10.7%) were included in this review. The reference search and expert interviews retrieved another 30 documents, of which 18 (60%) were included in this review. In total, 34 documents were included in this review. Figure 1 [34] shows the flowchart of the screening process and was prepared in accordance with the template provided by Page et al [34].

### Source Characteristics

**Figure 2:**
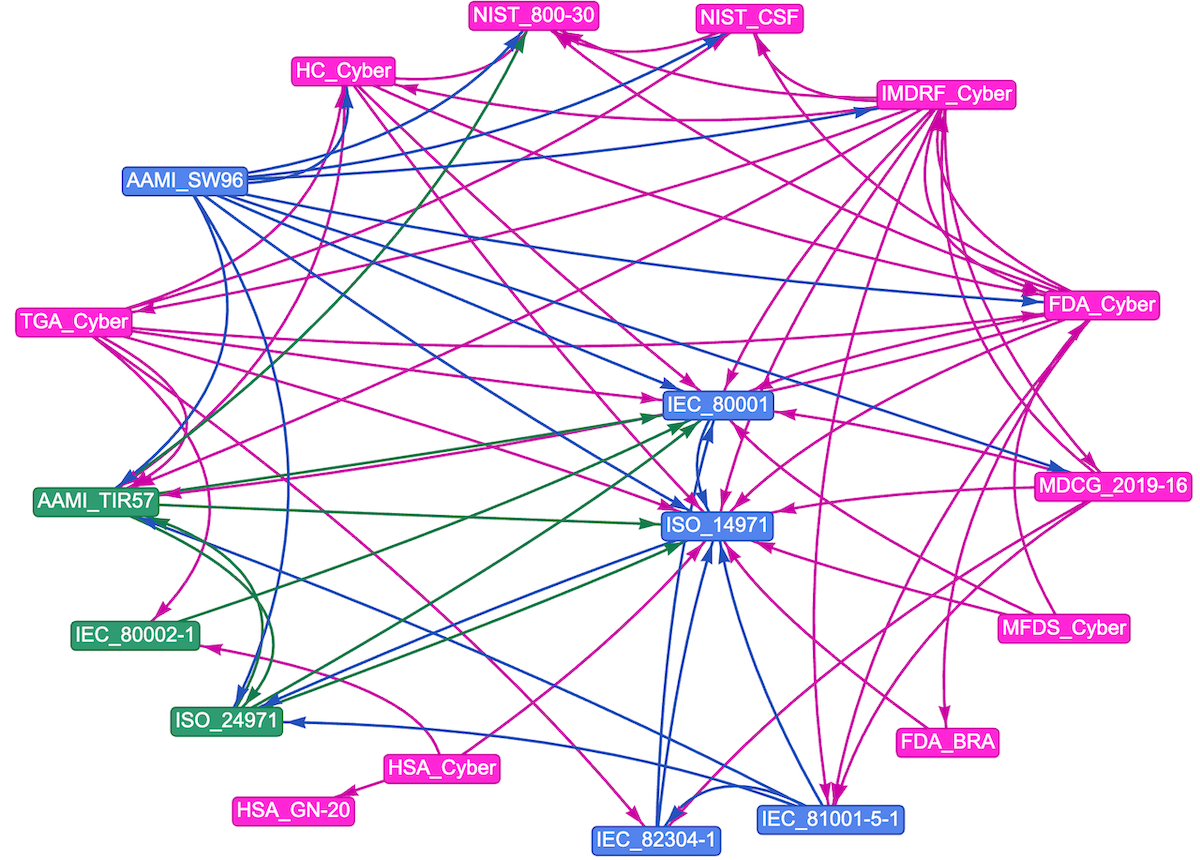
The relationships among the included documents. The nodes are color grouped by type. The edge color is inherited from the node it originates from. FDA_BRA: Benefit Risk Analysis Guidance from the Food and Drug Administration; FDA_Cyber: Cybersecurity Guidance from Food and Drug Administration HC_Cyber: Pre-market Requirements for Medical Device Cybersecurity; HSA_Cyber: Regulatory Guidelines for Software Medical Devices—A Life Cycle Approach; HSA_GN-20: GN-20: Guidance on Clinical Evaluation; IMDRF_Cyber: Cybersecurity Guidance from the International Medical Device Regulators Forum; MFDS_Cyber: Guideline on Review and Approval for Cybersecurity of Medical Devices (For industry); NIST_CSF: National Institute of Standards and Technology Cybersecurity Framework; TGA_Cyber: Cybersecurity Guidance from the Therapeutic Goods Administration.

Among the 34 documents included in the study were 4 (12%) regulations, 5 (15%) standards, 6 (18%) technical reports, and 19 (56%) guidance documents. In total, 6% (2/34) of the documents were applicable only in the EU, 18% (6/34) were applicable in the United States, 12% (4/34) were applicable in Australia, 6% (2/34) were applicable in Canada, 6% (2/34) were applicable in Singapore, 3% (1/34) were applicable in South Korea, 3% (1/34) were applicable in the United Kingdom, and 47% (16/34) were applicable internationally. A total of 15% (5/34) of the documents only described the concept of BRA without mentioning cybersecurity explicitly, 18% (6/34) described cybersecurity risks without mentioning the BRA, and 68% (23/34) described the intersection of both. In total, 24% (8/34) of the documents provided examples or methods on how to consider cybersecurity risks in the BRA. Table 1 provides an overview of the included documents, whereas Figure 2 shows a visual representation of the relationship between the documents. To improve readability, documents from the same source describing similar content were grouped together (eg, FDA_Cyber encompasses all Food and Drug Administration cybersecurity guidance and IEC_80001 encompasses all substandards of this standard family mentioned in this paper) and only the short titles of the included documents are used. A list with the URLs to the included documents can be found in Table S2 in Multimedia Appendix 1.

**Table 1:** Characteristics of the included documents. The table provides the complete name of the sources and the abbreviations used in this paper, if applicable. The year of publication and the year of the last update, if applicable, are provided.

Name	Abbreviation	Type	Organization	Year of publication	Year of last update	Description
Federal Food, Drug, and Cosmetic Act [19]	US_FD&C	Regulation	US House of Representatives	1938	2024	This is the law that grants the FDA^a^ the power to regulate MDs^b^. It establishes high-level rules for MDs and their approval process and defines requirements for many aspects of MDs, including cybersecurity and the BRA^c^.
Medical Devices Regulations (SOR/98-282) [23]	CA_MDR	Regulation	Parliament of Canada	1985	2024	This Canadian regulation defines basic principles and high-level requirements for the approval of MDs in Canada.
Therapeutic Goods (Medical Devices) Regulations 2002 [20]	AU_MDR	Regulation	Parliament of Australia	2002	2024	This Australian regulation defines basic principles and high-level requirements for the approval of MDs in Australia. The requirements are organized into 15 essential principles.
Regulation (EU^d^) 2017/745 of the European Parliament and of the Council of 5 April 2017, on MDs, amending Directive 2001/83/EC, Regulation (EC^e^) 178/2002, and Regulation (EC) 1223/2009 and repealing Council Directives 90/385/EEC and 93/42/EEC (text with EEA^f^ relevance) [18]	EU_MDR	Regulation	EU Parliament and Council	2017	2024	This European regulation came into force in 2017. It provides high-level rules and requirements for MDs that are or will be placed on the European market.
NIST^g^ SP^h^ 800-30 [35]	NIST_800-30	Guidance	NIST	2012	—^i^	This special publication by the NIST defines principles for how to conduct risk assessments for information systems. It is not specifically designed for MDs.
NIST Cybersecurity Framework [36]	NIST_CSF2.0	Guidance	NIST	2014	2024	This document by the NIST provides general guidance on cybersecurity risk management. It is not specifically designed for MDs.
Factors to Consider Regarding Benefit-Risk in Medical Device Product Availability, Compliance, and Enforcement Decisions [37]	FDA_BRA_AC & ED	Guidance	FDA	2016	2018	This document provides guidance for FDA staff and industry on the factors considered in the BRA regarding availability, compliance, and enforcement decisions.
Postmarket Management of Cybersecurity in Medical Devices [32]	FDA_Cyber_Post	Guidance	FDA	2016	2018	Provides guidance on managing cybersecurity vulnerabilities in MDs that are already on the market, emphasizing continuous monitoring and identifying and addressing cybersecurity threats to ensure safety and effectiveness throughout their life cycle.
GN-20: Guidance on Clinical Evaluation [38]	HSA_GN-20	Guidance	HSA^j^	2017	2022	This document provides guidance for the clinical evaluation required for the registration of MDs in Singapore.
Consideration of Uncertainty in Making Benefit-Risk Determinations in Medical Device Premarket Approvals, De Novo Classifications, and Humanitarian Device Exemptions [39]	FDA_BRA_Uncertainty	Guidance	FDA	2019	—	This document provides guidance on how the FDA considers uncertainty in MD premarket approvals and de novo classifications and describes factors influencing this uncertainty.
Factors to Consider When Making Benefit-Risk Determinations in Medical Device Premarket Approval and De Novo Classifications [40]	FDA_BRA	Guidance	FDA	2019	—	This document provides guidance on how the FDA conducts the BRA, explaining the factors considered and how they should be applied.
MDCG^k^ 2019-16, revision 1 [13]	MDCG_2019-16	Guidance	MDCG	2019	2020	Provides guidance for MD manufacturers on how to address the cybersecurity requirements stated by the MDR^l^. It outlines the necessary steps that manufacturers should take to ensure that their devices are secured against cyber threats throughout the product life cycle.
Pre-market Requirements for Medical Device Cybersecurity [22]	HC_Cyber	Guidance	Health Canada	2019	—	This document provides nonbinding guidance for the cybersecurity of MDs and about the information that should be provided when applying for a license in Canada.
Medical device cyber security guidance for industry [21]	TGA_Cyber_Dev	Guidance	TGA^m^	2019	2022	This document provides guidance on how to comply with the essential principles for MDs in Australia, focusing on cybersecurity.
Medical device cyber security information for users [41]	TGA_Cyber_User	Guidance	TGA	2019	2022	This document provides recommendations for users of MDs regarding cybersecurity practices. It emphasizes the importance of users and operating environments for secure MDs.
Principles and Practices for Medical Device Cybersecurity [42]	IMDRF_Cyber	Guidance	IMDRF^n^	2020	—	It provides principles and recommendations regarding the cybersecurity of MDs for all stakeholders and emphasizes the relevance of a risk management process for cybersecurity risks.
Regulatory Guidelines for Software Medical Devices—A Life Cycle Approach [43]	HSA_Cyber	Guidance	HSA	2020	2024	The guideline details the regulatory requirements for software in MDs or as an MD over the product’s life cycle. This includes aspects of cybersecurity, including risk management.
Guideline on Review and Approval for Cybersecurity of Medical Devices (For industry) [44]	MFDS_Cyber	Guidance	MFDS^o^	2020	—	This nonbinding guideline for manufacturers provides an overview of the requirements and how they can be met for the approval of MDs in South Korea.
Software and AI as a Medical Device Change Programme [45]	MHRA_SaMD	Guidance	MHRA^p^	2021	2023	This document provides an overview of upcoming changes to the regulation of MDs in the United Kingdom regarding software and AI^q^. It does not deliver clear guidance in its current state.
Complying with the Essential Principles on the safety and performance of medical devices [46]	TGA_Safety	Guidance	TGA	2022	2024	This guidance describes how the 15 essential principles for MD approval in Australia defined in the “Therapeutic Goods (Medical Devices) Regulations 2002” should be met and addressed.
Cybersecurity in Medical Devices: Quality System Considerations and Content of Premarket Submissions [22]	FDA_Cyber_Pre	Guidance	FDA	2023	—	Provides guidance for MD manufacturers on addressing cybersecurity in their premarket submissions, emphasizing the importance of integrating robust cybersecurity measures throughout the device’s life cycle to ensure safety and effectiveness.
Principles and Practices for Software Bill of Materials for Medical Device Cybersecurity [47]	IMDRF_SBOM	Guidance	IMDRF	2023	—	Outlines principles and recommendations for the implementation of an SBOM^r^ to enhance cybersecurity for MDs.
Principles and Practices for the Cybersecurity of Legacy Medical Devices [48]	IMDRF_LegMD	Guidance	IMDRF	2023	—	Provides principles and recommendations for maintaining the cybersecurity of legacy MDs.
IEC^s^ 82304-1:2016 [49]	IEC_82304-1	Standard	IEC	2016	—	This document provides requirements for safety and security over the entire life cycle of MDs that constitute or contain software.
ISO^t^ 14971:2019 [11]	ISO_14971	Standard	ISO	2019	—	Describes how the risk management process for MDs should be conducted by MD manufacturers.
IEC 80001-1:2021 [50]	IEC_80001-1	Standard	IEC	2021	—	This standard describes the requirements for different stakeholders in the risk management process of MDs connected to a health care infrastructure. It covers multiple risk types, including cybersecurity risks.
IEC 81001-5-1:2021 [51]	IEC_81001-5-1	Standard	IEC	2021	—	This standard defines life cycle requirements for MDs regarding cybersecurity, including best practices and security risk management.
ANSI^u^/AAMI^v^ SW96:2023 [52]	AAMI_SW96	Standard	AAMI	2023	—	This standard applies the framework provided within ISO 14971:2019 to security risk management.
IEC 80002-1:2009 [53]	IEC_80002-1	Technical report	IEC	2009	—	This technical report details how to apply the risk management framework of ISO 14971 to software as an MD or in MDs.
IEC/TR^w^ 80001-2-2:2012 [54]	IEC_80001-2-2	Technical report	IEC	2012	—	This document provides a framework for disclosing security-related capabilities and risks associated with the risk management of MDs connected to IT networks.
IEC/TR 80001-2-1:2012 [55]	IEC_80001-2-1	Technical report	IEC	2012	—	This document provides guidance on how to implement the risk management process described in IEC 80001-1:2021.
ISO/TR 80001-2-7:2015 [56]	ISO_80001-2-7	Technical report	ISO	2015	—	This document provides guidance on how to assess MD conformity with IEC 80001-1:2021.
AAMI TIR57:2016/(R)2023 Principles for medical device security—Risk Management [57]	AAMI_TIR57	Technical report	AAMI	2016	2023	Provides guidance on methods for performing cybersecurity risk management for MDs incorporating principles from ISO 14971:2019 and IEC 80001-1:2021.
ISO TR 24971:2020 [12]	ISO_24971	Technical report	ISO	2020	—	Provides guidance on the application of ISO 14971:2019.

^a^FDA: Food and Drug Administration.

^b^MD: medical device.

^c^BRA: benefit-risk analysis.

^d^EU: European Union.

^e^EC: European Community.

^f^EEA: European Economic Area.

^g^NIST: National Institute of Standards and Technology.

^h^SP: Special Publications.

^i^The document was not updated.

^j^HSA: Health Sciences Authority.

^k^MDCG: Medical Device Coordination Group.

^l^MDR: Medical Device Regulation.

^m^TGA: Therapeutic Goods Administration.

^n^IMDRF: International Medical Device Regulators Forum.

^o^MFDS: Ministry of Food and Drug Safety.

^p^MHRA: Medicines and Healthcare Products Regulatory Agency.

^q^AI: artificial intelligence.

^r^SBOM: software bill of materials.

^s^IEC: International Electrotechnical Commission.

^t^ISO: International Organization for Standardization.

^u^ANSI: American National Standards Institute.

^v^AAMI: Association for the Advancement of Medical Instrumentation.

^w^TR: Technical Report.

### Regulations in the EU, the United States, Canada, and Australia

The scope and requirements for MD manufacturers in the EU are determined at a high level by the Regulation (EU) 2017/745 of the European Parliament and of the Council on MDs (EU_MDR) [18] and by the Regulation (EU) 2017/746 of the European Parliament and of the Council on in vitro diagnostic MDs [58]. The latter was excluded from this scoping review. The provided rules are further defined by guidance documents issued by the MDCG or by referring to existing harmonized standards developed or adapted from existing international standards by a European standard organization [18]. In the United States, the FD&C Act defines foundational requirements for MDs and empowers the FDA to establish detailed rules for MDs and enforce them [19]. The situation is similar in the other regions analyzed. While the fundamental regulation is usually passed by the legislature, it is specified by executive organizations and authorities such as the TGA in Australia or the HSA in Singapore [20,21,23,43,45,59].

In the EU, the EU_MDR addresses BRA requirements in multiple sections, requiring weighing all known risks of a device against its benefits to the patient [18] without further specifying the types of risks. While not mentioned explicitly, it is generally assumed that cybersecurity risks should be considered. Cybersecurity risks are not mentioned in detail; instead, the EU_MDR defines 2 broad requirements using the terms “information security” and “IT security”: MDs that include software or are software must be developed following the state of the art and must have protective measures against unauthorized access [18]. This state of the art is not further defined.

In the United States, the FD&C Act section 515(a) states that the effectiveness and safety of an MD are determined by “weighing any probable benefit to health from the use of the device against any probable risk of injury or illness from such use” [19]. High-level cybersecurity requirements are defined in section 524B of the FD&C Act to monitor and address postmarket vulnerabilities, ensure device and system security, provide software updates and a software bill of materials (SBOM), and comply with additional regulations for cybersecurity assurance [19]. Similarly to the EU, the intersection of cybersecurity risks and BRA is not mentioned explicitly within the regulation, but the wording in both the EU_MDR and the FD&C Acts suggests a consideration.

The Canadian Medical Device Regulations require conducting a BRA to determine whether a product is safe and should be put on the market. It is not specified which risks should be considered or how the BRA should be conducted. Cybersecurity is not mentioned [23].

In the Australian Therapeutic Goods (Medical Devices) Regulations (AU_MDR) 2002, the requirement of the BRA and high-level cybersecurity requirements are mentioned separately. The performance of a BRA is prescribed in “Essential Principle 6,” with the requirement that the benefits of an MD must outweigh any undesirable effects arising from its use [20]. While cybersecurity is not mentioned directly, additional guidance clarifies that cybersecurity risks are within the scope of this principle [21]. Additional cybersecurity requirements are defined in “Essential Principle 12,” including the implementation of a risk management process [20].

### Guidelines for the BRA and Cybersecurity

Among the 34 documents, we identified 19 (56%) guidelines currently used within the included regions that describe the concept of the BRA or cybersecurity risks. Table 2 provides an overview of the identified regulations, guidelines and standards, and their recommendations for including cybersecurity risks in the BRA.

One guideline was issued by the MDCG, expanding and framing the cybersecurity requirements provided in the EU_MDR. In addition to implementing cybersecurity measures, manufacturers must establish a process for managing cybersecurity risks that considers the device’s safety, security, and effectiveness. The residual security risks (which could include cybersecurity ones) should then be considered in a newly named concept called the “security benefit-risk analysis” [13]. However, it remains unclear when the BRA should be performed as the corresponding passage is ambiguous: the BRA could be divided into subcategories identified in the risk assessment (eg, cybersecurity risks, electromagnetic risks, and usability risk), or all risks, including cybersecurity ones, may be considered within the overall BRA [13]. Further details or guidance about the method to use are not provided.

A total of 9% (3/34) of the documents were issued by the FDA, providing nonbinding but recommended guidance on how to conduct a BRA for MD approval in the United States, including practical examples [37,39,40]. The qualitative method described in those guidance documents is known as the FDA’s BRF and could be adapted for the consideration of cybersecurity risks; however, in its current state, it provides no specific guidance on how this can be done. The other 2 FDA guidelines describe cybersecurity requirements for MDs, mentioning the relevance of cybersecurity risks for BRA multiple times [22,32] and partly referring to the FDA’s BRF.

The cybersecurity guidance provided by Health Canada recommends incorporating cybersecurity risks into the risk management process, whereas the BRA is only mentioned superficially [59].

One guidance document about the clinical evaluation of MDs issued by the HSA describes the BRA as an ongoing process that includes any risks associated with using the MD without mentioning cybersecurity directly [38]. Another HSA guidance document about cybersecurity recommends that the framework set out in the International Organization for Standardization (ISO) standard 14971 for cybersecurity risk management be followed, which includes the BRA as an integral step of this process [43].

The MHRA guidance on cybersecurity acknowledges the relevance of cybersecurity and provides a road map for future developments without defining cybersecurity requirements or mentioning the BRA in its current state [45].

A TGA guidance document describes the implementation of the 15 “Essential Principles” of the AU_MDR, containing recommendations for both the BRA and cybersecurity [46]. The BRA is described as a holistic approach that assures that the benefits from the use of an MD outweigh any undesirable effects while providing no operational method [46]. The cybersecurity requirements, which are described at a high level in this guidance, are further detailed in 2 additional guidance documents [21,41]. These recommend a continuous risk assessment and management over the MD’s life cycle that includes cybersecurity risks and the consideration of cybersecurity risks in the BRA without providing a detailed methodology or description of the impact of cybersecurity on the BRA [21,41].

The MFDS guidance defines high-level requirements for the cybersecurity risk management process without mentioning the BRA [44].

In total, 9% (3/34) of the guidelines were issued by the IMDRF, a joint group of multiple regulatory authorities, including the European Commission and the FDA. While the provided documents are not legally binding, they provide a good overview of regulatory practices and are acknowledged as part of the overall regulatory state of the art by the FDA as well as by EU regulatory bodies [13,22]. These 3 guidelines describe cybersecurity recommendations for MDs, acknowledging the need to conduct a BRA that considers cybersecurity risks [42,47,48].

A total of 6% (2/34) of the guidelines were issued by the National Institute of Standards and Technology (NIST) but are referenced in multiple guidance documents originating from sources outside the United States [21,42,59]. The NIST_800-30 provides guidance on the implementation of a risk management process, describing different methods while addressing the concept of the BRA only slightly [35]. Similarly, the NIST Cybersecurity Framework provides guidance for cybersecurity risk management with a focus on organizations but does not describe the BRA at all [36].

**Table 2:** The consideration of cybersecurity risks in the benefit-risk analysis (BRA) as defined in different regulations, guidelines, and standards.

Name	Mention of BRA	Mention of cybersecurity	Provides a method or example	Intersection between BRA and cybersecurity
US_FD&C^a^	Yes	Yes	No	The FD&C Act addresses the BRA and cybersecurity independently. However, the broadly defined risks in the BRA include safety risks that could be caused by cybersecurity vulnerabilities, ensuring comprehensive risk management without explicitly mentioning cybersecurity.
CA_MDR^b^	Yes	No	No	The CA_MDR requires conducting a BRA to determine whether a product is safe and effective. It acknowledges the uncertainty that lies in this process but provides no further detail on methods. It does not mention cybersecurity.
AU_MDR^c^	Yes	Yes	No	The AU_MDR mentions cybersecurity requirements and the BRA independently in its “Essential Principles.” Principle 6 defines that the benefits of an MD^d^ must outweigh any undesirable effects arising from its use. Guidance documents by the TGA^e^ clarify that cybersecurity is within the scope of this principle. No detailed method is provided. In principle 12, the document defines requirements for the cybersecurity of MDs, including the implementation of a risk management process.
EU_MDR^f^	Yes	Yes	No	The EU_MDR addresses the BRA and cybersecurity independently. However, the broadly defined risks in the BRA include safety risks that could be caused by cybersecurity vulnerabilities, ensuring comprehensive risk management without explicitly mentioning cybersecurity.
NIST_800-30	Yes	Yes	No	The document provides guidance on the cybersecurity risk assessment process and describes the benefits and problems of qualitative, quantitative, and semiquantitative methods, acknowledging the limitations of personal judgment and uncertainties of the process. Due to the non–MD-specific nature of the document, the concept of a BRA is only slightly addressed, whereas the main focus is on cost and benefit trade-offs.
NIST_CSF2.0^h^	No	Yes	No	The document provides a framework for cybersecurity risk management with a focus on organizations. The BRA is not mentioned.
FDA_BRA_AC&ED^i^	Yes	No	Yes (not specifically designed for cybersecurity)	This document supplements the FDA’s^j^ BRA framework by incorporating additional benefits and risks. It specifically describes the impact of availability, compliance, and enforcement decisions on the BRA.
FDA_Cyber_Post^k^	Yes	Yes	No	This guidance document advises manufacturers to monitor, identify, and address cybersecurity vulnerabilities and exploits as part of their postmarket management of MDs. It also recommends evaluating residual risks, the results of the BRA, and any risks introduced through remediation efforts as part of the Postmarket Cybersecurity Program after detecting a vulnerability.
HSA_GN-20^l^	Yes	No	No	This guidance defines an important objective of clinical evaluation as determining whether the risks associated with the use of the MD are acceptable when weighed against the benefits for the patient. This BRA is also seen as an ongoing process that includes any risks associated with the use of the MD without mentioning cybersecurity directly.
FDA_BRA_Uncertainty^m^	Yes	No	No	This guidance document addresses the inherent uncertainty in MDs’ premarket decision-making regarding their benefits and risks. It outlines the need to consider this uncertainty in the BRA and recommends collecting postmarket data to address and reduce this uncertainty over an MD’s life cycle.
FDA_BRA^n^	Yes	No	Yes (not specifically designed for cybersecurity)	This guidance document states that the BRA can be conducted using both clinical and nonclinical data, recognizing that some MDs’ attributes cannot be tested using clinical methods. It emphasizes that clinical benefits are usually measured directly through factors such as magnitude, probability, and duration. In the BRA, all risks must be considered and weighed against the clinical benefits, both based on the totality of the evidence. The document also highlights the importance of incorporating patient perspectives and accounting for uncertainty. It provides an assessment tool similar to a checklist and examples and notes that the BRA is an essential step that should always be conducted following the risk assessment and management processes that are outlined in ISO^o^ standard 14971:2019.
MDCG_2019-16^p^	Yes	Yes	Yes	This guidance document emphasizes the need to consider the relationship between safety and security in the context of risk. It highlights that security risks can be caused by both weak and restrictive security measures. Rather than conducting a BRA for each individual security risk, an overall BRA should be executed based on the device’s intended use and the potential impacts on safety and performance using the safety risk assessment, which includes security hazard categories. In addition, the original security BRA should be updated with PMS^q^ data.
HC_Cyber^r^	Yes	Yes	No	In this document, cybersecurity is seen as an integral component of an MD over its complete life cycle. The overlap between security and safety is acknowledged, and it is recommended to incorporate cybersecurity into the risk management process. The relevance of the BRA is only mentioned superficially in the introduction without providing further details on the impact of cybersecurity risks.
TGA_Cyber_Dev^s^	Yes	Yes	No	This document recommends a continuous risk assessment and management over the MD’s life cycle that includes cybersecurity risks. Devices should be secure by design and by default to minimize risks to patients. The overlap of security and safety is acknowledged. It is stated that, to comply with principle 6 of the Therapeutic Goods (Medical Devices) Regulations 2002, cybersecurity risks must be considered in the BRA. The document does not provide a detailed methodology or description of the impact of cybersecurity on the BRA. Instead, it offers general recommendations, such as maintaining an SBOM^t^, and suggests that new features could potentially increase cybersecurity risks but that they should also provide benefits.
TGA_Cyber_User^u^	Yes	Yes	No	This document emphasizes the role of users to maintain security in MDs. The BRA is mentioned in the context of communication activities, which should include discussions on the benefits of a device versus its cybersecurity risks.
IMDRF_Cyber^v^	Yes	Yes	No	This guidance document emphasizes the need to assess the impact of security risk mitigation measures on the management of other risks (eg, considering the benefits and risks associated with deploying updates). HCPs^w^ are encouraged to conduct a BRA of manufacturers’ proposed mitigations before implementing them. According to this document, a cybersecurity-informed BRA is an ongoing process.
HSA_Cyber^x^	Yes	Yes	No	This document recommends the implementation of an ongoing risk management process following ISO standard 14971, which also takes cybersecurity risks into account. The overlap of security and safety risks is acknowledged. The BRA is mentioned in the context of clinical evaluation and new risks arising from the use of a device without specifying a method or describing the impact of cybersecurity risks on the BRA.
MFDS_Cyber^y^	No	Yes	No	This document acknowledges an overlap of security and safety risks and defines high-level requirements for the cybersecurity risk management process. The concept of a BRA is not mentioned.
MHRA_SaMD^z^	No	Yes	No	The relevance of cybersecurity risks and a gap in the current regulation are recognized. A road map for future developments is presented but without providing details on risk management in the area of cybersecurity or on the BRA.
TGA_Safety^aa^	Yes	Yes	No	In line with principle 6 of the Therapeutic Goods (Medical Devices) Regulations 2002, this guidance emphasizes the relevance of the BRA. The BRA is described as a holistic method that assures that the benefits from the use of an MD outweigh any undesirable effects. A process for risk assessment and risk management must be implemented throughout the life cycle of the product. The impact of cybersecurity risks on the BRA is not described in detail, and no method is given for how a BRA must be performed.
FDA_Cyber_Pre^ab^	Yes	Yes	No	This guidance document emphasizes the unpredictable nature of cybersecurity risks, which cannot be easily assessed or quantified using historical data or modeling. Thus, qualitative methods could also be used, which is similarly acknowledged in the most recent version of ISO standard 14971:2019. This document also acknowledges the interconnected yet distinct nature of safety and security risk management. Manufacturers should assess identified risks based on the level of risk posed by the device and its operational system, with continuous risk identification throughout the device life cycle. Cybersecurity should be integrated into the device from the beginning. Responses to security events should consider the BRA to determine the appropriateness of actions given that updates may limit device availability. In addition, this document recognizes the challenges in updating devices already on the market.
IMDRF_SBOM^ac^	Yes	Yes	No	This guidance document highlights that providing an SBOM enables regulators to perform a more comprehensive BRA. It aids in estimating and addressing the impact of threats, vulnerabilities, and exploits, thereby enhancing overall risk management.
IMDRF_LegMD^ad^	Yes	Yes	No	This guidance document advises HCPs and MD manufacturers to monitor the risk profile of devices throughout their life cycle. HCPs should perform regular clinical BRAs comparing the risks of the use of legacy devices beyond their EOS^ae^ date with acquiring new or upgraded devices.
IEC_82304-1 standard^af^	No	Yes	No	The standard defines that a risk management process should be in place to fulfill its risk-benefit approach. The risk assessment should also include the network environment. The impact of cybersecurity risks on risk management is only described at a high level without providing details on the BRA.
ISO_14971 standard^ag^	Yes	Yes	Yes (not specifically designed for cybersecurity)	ISO 14971:2019 acknowledges that quantitative data are often unavailable for cybersecurity risks, allowing for qualitative risk estimation. The standard specifically includes risks related to data and system security within its scope. It highlights that breaches in data and system security can lead to harm, such as loss of data, uncontrolled access to data, corruption or loss of diagnostic information, or software corruption resulting in device malfunction. If a residual risk is deemed unacceptable based on the risk management plan’s criteria and further risk control is impractical, manufacturers may review data and literature to determine whether the benefits of the intended use outweigh this residual risk. In addition, a general BRA of all residual risks should be conducted by weighing them against the overall benefits provided by the MD’s intended use. This overall residual risk could be stronger than each individual risk.
IEC_80001-1 standard^ah^	Yes	Yes	Yes	This standard specifies that, if risks cannot be reduced to an acceptable level through risk management, a BRA should be conducted. When no suitable risk control measures are possible, a holistic BRA should be performed, weighing the overall residual risk against the system benefit. The analysis should involve judgment by experienced and knowledgeable individuals considering technical, clinical, regulatory, economic, sociological, and political contexts. It also recommends conducting a general BRA weighing all unaccepted risks against the net clinical benefit of deployment, aligning with the guidance in ISO TR^ai^ 24971:2020.
IEC_81001-5-1 standard^aj^	No	Yes	No	This standard mandates that handling residual security risks should be done in cooperation with product risk management. It also requires considering the impacts on safety caused by the degradation of security over time.
AAMI_SW96 standard^ak^	Yes	Yes	Yes	If a security residual risk is deemed unacceptable, a BRA should be performed. Manufacturers should balance the residual security risk against the benefits provided by the device’s design capabilities or security controls. The overall BRA should consider all security residual risks as well as the impact of the implementation of the device into the IT infrastructure, intersecting with the requirements defined in IEC 80001-1:2021.
IEC_80002-1^al^	Yes	Yes	No	This document has a high overlap with ISO 14971 as it describes the application of ISO 14971 for software. The BRA is described as an integral part of the risk management process for all residual risks. Cybersecurity is mentioned as a relevant risk, whereas further details of its impact on the BRA are not provided.
IEC_80001-2-2	No	Yes	No	This technical report emphasizes that residual cybersecurity risks should be considered in the BRA.
IEC_80001-2-1	Yes	Yes	No	This technical report specifies that, if risks cannot be reduced to an acceptable level through the risk management process, the device should be changed or the risk should be outweighed by the expected benefits, determined in a BRA.
ISO_80001-2-7	Yes	Yes	No	This technical report specifies that individual and overall residual risks should be assessed for acceptability. If the required risk reduction is impractical, the responsible organization must conduct and document a BRA of the residual risk. In addition, when the residual risk remains unacceptable, a BRA should be conducted to weigh the overall residual risk against the health benefits emerging from incorporating the MD into the IT network.
AAMI_TIR57^am^	Yes	Yes	Yes	This technical report advises that, if a security residual risk is deemed unacceptable, a BRA should be performed. Manufacturers should balance the residual security risk against the benefits provided by the device’s design capabilities or security controls. It warns against using “security by obscurity” as a risk reduction strategy. Security risks impacting safety must be evaluated in the safety risk assessment following ISO 14971:2019. Effective communication with patients and HCPs is crucial, ensuring that they understand how to manage residual risk without providing attackers with a blueprint. Manufacturers should also balance usability, device safety, and security to ensure appropriate security controls for intended users and connected systems.
ISO_24971^an^	Yes	Yes	Yes (not specifically designed for cybersecurity)	This technical report provides examples of cybersecurity-related safety risks and emphasizes the importance of evaluating security risks by considering confidentiality, integrity, and availability in the context of the device’s intended use. It states that the BRA is used to determine whether an individual residual risk is outweighed by the expected benefits of the device’s intended use. Manufacturers are encouraged to consider technical, regulatory, economic, and sociological contexts in their risk management decisions, acknowledging that implementing risk control measures might introduce new risks or exacerbate existing ones. The report emphasizes the complexity of directly comparing benefits and risks and suggests that the overall residual risk should be viewed from a broad perspective, ensuring that all identified hazardous situations have been evaluated and risks have been reduced to an acceptable level or accepted based on a BRA.

^a^FD&C: Federal Food, Drug, and Cosmetic Act.

^b^CA_MDR: Canadian Medical Device Regulations.

^c^AU_MDR: Australian Therapeutic Goods (Medical Devices) Regulations.

^d^MD: medical device.

^e^TGA: Therapeutic Goods Administration.

^f^EU_MDR: European Union Medical Device Regulation.

^g^NIST_800-30: National Institute of Standards and Technology Special Publication 800-30.

^h^NIST_CSF2.0: National Institute of Standards and Technology Cybersecurity Framework.

^i^FDA_BRA_AC&ED: Factors to Consider Regarding Benefit-Risk in Medical Device Product Availability, Compliance, and Enforcement Decisions.

^j^FDA: Food and Drug Administration.

^k^FDA_Cyber_Post: Postmarket Management of Cybersecurity in Medical Devices.

^l^HSA_GN-20: GN-20: Guidance on Clinical Evaluation.

^m^FDA_BRA_Uncertainty: Consideration of Uncertainty in Making Benefit-Risk Determinations in Medical Device Premarket Approvals, De Novo Classifications, and Humanitarian Device Exemptions.

^n^FDA_BRA: Factors to Consider When Making Benefit-Risk Determinations in Medical Device Premarket Approval and De Novo Classifications.

^o^ISO: International Organization for Standardization.

^p^MDCG_2019-16: Medical Device Coordination Group Document 2019-16–Guidance on Cybersecurity for medical devices.

^q^PMS: postmarket surveillance.

^r^HC_Cyber: Pre-market Requirements for Medical Device Cybersecurity.

^s^TGA_Cyber_Dev: Medical device cybersecurity guidance for industry.

^t^SBOM: software bill of materials.

^u^TGA_Cyber_User: Medical device cybersecurity information for users.

^v^IMDRF_Cyber: Principles and Practices for Medical Device Cybersecurity.

^w^HCP: health care provider.

^x^HSA_Cyber: Regulatory Guidelines for Software Medical Devices—A Life Cycle Approach.

^y^MFDS_Cyber: Guideline on Review and Approval for Cybersecurity of Medical Devices (for industry).

^z^MHRA_SaMD: Software and AI as a Medical Device Change Programme.

^aa^TGA_Safety: Complying with the Essential Principles on the safety and performance of medical devices.

^ab^FDA_Cyber_Pre: Cybersecurity in Medical Devices: Quality System Considerations and Content of Premarket Submissions.

^ac^IMDRF_SBOM: Principles and Practices for Software Bill of Materials for Medical Device Cybersecurity.

^ad^IMDRF_LegMD: Principles and Practices for the Cybersecurity of Legacy Medical Devices.

^ae^EOS: end of service.

^af^IEC_82304-1 standard: International Electrotechnical Commission Standard 82304-1:2016 Health software Part 1: General requirements for product safety.

^ag^ISO_14971 standard: International Organization for Standardization Standard 14971:2019 Medical devices—Application of risk management to medical devices.

^ah^IEC_80001-1 standard: International Electrotechnical Commission Standard IEC 80001-1:2021 Application of risk management for IT-networks incorporating medical devices; Part 1: Safety, effectiveness and security in the implementation and use of connected medical devices or connected health software.

^ai^TR: Technical Report.

^aj^IEC_81001-5-1 standard: International Electrotechnical Commission Standard 81001-5-1:2021 Health software and health IT systems safety, effectiveness and security; Part 5-1: Security — Activities in the product life cycle.

^ak^AAMI_SW96 standard: American National Standards Institute and Association for the Advancement of Medical Instrumentation Standard SW96:2023; Standard for medical device security—Security risk management for device manufacturers.

^al^IEC_80002-1: International Electrotechnical Commission Standard 80002-1:2009 Medical device software; Part 1: Guidance on the application of ISO 14971 to medical device software.

^am^AAMI_TIR57: Association for the Advancement of Medical Instrumentation Technical Report TIR57:2016/(R)2023; Principles for medical device security—Risk management.

^an^ISO_24971: International Organization for Standardization Technical Report 24971:2020 Medical devices — Guidance on the application of ISO 14971.

### Standards for the BRA and Cybersecurity

Among the 34 documents, 5 (15%) were standards and 6 (18%) were technical reports. All of them describe cybersecurity considerations for the BRA process. The ISO 14971:2019 standard explicitly mentions that security risks should be considered in the risk assessment and the BRA [11] as they can lead to harm [12]. This standard is the recognized consensus standard of the FDA for the risk management of MDs and was harmonized for the EU as the EN ISO 14971:2019 standard [60]. It also lists basic requirements for the BRA, mandating an evaluation of whether a device’s benefits outweigh its residual risks and necessary modifications if the benefits do not justify the risks [11]. The ISO Technical Report 24971:2020 standard, a technical report on how to implement ISO 14971:2019, provides examples and detailed guidance on which risks to consider within the security risk management process. In line with ISO 14971:2019, it proposes that the overall residual risk should be viewed from a broad perspective considering all residual risks, ensuring that all identified hazardous situations have been evaluated and the risks have been reduced to an acceptable level or accepted based on a BRA [12].

The International Electrotechnical Commission (IEC) 80001-1:2021 standard and the associated technical reports describe that, if single individual risks cannot be reduced to an acceptable level through mitigation measures in the risk management process, a BRA should be conducted for each of those risks individually [50]. In addition, when residual risks remain, the aggregated residual risk should be weighed against the benefits emerging from the deployment of an MD [50]. The IEC 81001-5-1:2021 standard mandates that handling residual security risks should be done in cooperation with product risk management without mentioning a BRA explicitly [51].

The Association for the Advancement of Medical Instrumentation (AAMI) TIR57 standard is referred to in multiple cybersecurity-related FDA guidance documents and explains in detail how information security risk management for MDs should be performed and how the identified risks should be considered in the BRA [57]. It states that, if a security-related residual risk is assessed as unacceptable, a BRA should be performed. Manufacturers should balance the residual risk against the benefits provided by the device’s design capabilities or security controls. This report also provides an example of an unacceptable cybersecurity-related residual risk and how this affects the BRA outcome: an unacceptable risk of exposing personally identifiable information should not be justified solely because the device provides life-saving therapy. Instead, the benefit of storing the information on the device should be weighed against the risk of compromising confidentiality [57].

The FDA-recognized AAMI SW96 standard applies the risk management framework provided in the ISO 14971:2019 standard to security risks and defines requirements similar to those of the AAMI TIR57 standard [52]. In addition, it provides an example of how a security benefit could outweigh a security risk and adds an infrastructure view to an overall security BRA: not only the device itself must be considered but also the impact of the implementation of the device into the IT infrastructure, intersecting with the requirements defined in the IEC 80001-1:2021 standard [52].

The IEC 80002-1:2009 standard describes the application of ISO standard 14971 for MD software and acknowledges the relevance of cybersecurity risks without adding information to the framework set out in ISO standard 14791 [53].

The IEC 82304-1:2016 standard describes the implementation of risk management that considers cybersecurity risks as relevant for its benefit-risk approach. Further details on the BRA are not provided [49].

### Recommendations and Best Practices

Within the identified documents, several recommendations and best practices on cybersecurity risks in a BRA for manufacturers were identified. Table 3 provides an overview of those recommendations, including the provision of an SBOM [19,21,43,47]; recommendations for a BRA that considers the overall residual risks, including cybersecurity-related ones in the context of clinical benefits [11-13,21,37,39,40,46], and is conducted on a regular basis as part of postmarket surveillance (PMS) activities [13,21,32,38,43,51,59]; recommendations for considering the ISO 14971 framework [21,22,43,52,53,57]; and general development requirements to minimize the risk to patients [13,18,20-22].

**Table 3:** Recommendations on how to conduct a benefit-risk analysis (BRA) that considers cybersecurity risks. In addition to the recommendation and its description, the phase of the medical device (MD) life cycle in which a recommendation is relevant for the BRA is provided.

Recommendation	Document it was included in	Description	Phase of the MD life cycle
Prepare an SBOM^a^.	IMDRF_SBOM^b^, HSA_Cyber^c^, TGA_Cyber_Dev^d^, and US_FD&C^e^	Manufacturers should provide an SBOM to facilitate the BRA for regulators. The SBOM should be updated on a regular basis.	Premarket and postmarket phases
A BRA should always be conducted.	TGA_Safety^f^, AU_MDR^g^, MDCG_2019-16^h^, and FDA_BRA^i^	The BRA should always be conducted as part of the overall risk management process and not only if unacceptable residual risks remain as a combination of risks could have a higher impact than each individual risk.	Premarket phase
An overall BRA should be conducted.	MDCG_2019-16, FDA_BRA, FDA_BRA_Uncertainty^j^, FDA_BRA_AC&ED^k^, ISO_14971^l^, ISO_24971^m^, TGA_Safety, and TGA_Cyber_Dev	Instead of conducting a BRA for each cybersecurity risk individually, an overall BRA should be conducted considering the overall residual risks, including cybersecurity-related ones, in the context of clinical benefits.	Premarket and postmarket phases
The entire IT infrastructure should be considered.	TGA_Cyber_Dev, IEC_82304-1, and MDCG_2019-16	In the BRA, not only the cybersecurity risks for the individual device must be considered but also the impact of the implementation of the device into the IT infrastructure as the device might introduce new vulnerabilities that influence all other devices in the network.	Premarket and postmarket phases
Effective communication of residual risks	AAMI_TIR57^o^, TGA_Cyber_User^p^, and IMDRF_Cyber^q^	If residual risks remain, manufacturers should maintain good communication with patients and health care providers, describing those risks without delivering a blueprint for an attacker.	Postmarket phase
Security event responses and updates should be guided by a BRA.	FDA_BRA_AC&ED, FDA_Cyber_Pre^r^, FDA_Cyber_Post^s^, and IMDRF_Cyber	The response to security events and the deployment of updates should be guided by a BRA as both could cause new risks for patients.	Postmarket phase
The cybersecurity BRA should be part of postmarket surveillance activities.	HC_Cyber^t^, HSA_Cyber, HSA_GN-20^u^, TGA_Cyber_Dev, FDA_Cyber_Post, MDCG_2019-16, and IEC_81001-5-1	As previously unknown vulnerabilities could emerge in cMDs^v^ that can change the benefit-risk ratio, the BRA must be conducted regularly as part of postmarket surveillance activities. For this, a continuous threat analysis and response plan is required.	Postmarket phase
A device’s EOS^w^ should be considered.	TGA_Cyber_Dev, IMDRF_LegMD^x^, and HSA_Cyber	For devices after the EOS, the BRA conducted by the health care provider should consider the benefits and risks of using old devices versus acquiring new ones.	Postmarket phase
The framework set out in ISO^y^ standard 14971 should be considered.	HSA_Cyber, TGA_Cyber_Dev, IEC_80002-1, AAMI_TIR57, AAMI_SW96, and FDA_Cyber_Pre	ISO standard 14971 provides a standardized and recognized framework for risk management, of which the BRA is a part. ISO standards 14971 and 24971 state that the framework could also be used for cybersecurity risks.	Premarket and postmarket phases
Devices should be developed to minimize risks.	TGA_Cybyer_Dev, MDCG_2019-16, AU_MDR, EU_MDR^z^, and FDA_Cyber_Pre	To minimize the risk to patients, devices should follow the state of the art; be secure by design; and be able to respond to future, as yet unknown threats.	Premarket phase

^a^SBOM: software bill of materials.

^b^IMDRF_SBOM: Principles and Practices for Software Bill of Materials for Medical Device Cybersecurity.

^c^HSA_Cyber: Regulatory Guidelines for Software Medical Devices—A Life Cycle Approach.

^d^TGA_Cyber_Dev: Medical device cybersecurity guidance for industry.

^e^FD&C: Federal Food, Drug, and Cosmetic Act.

^f^TGA_Safety: Complying with the Essential Principles on the safety and performance of medical devices.

^g^AU_MDR: Australian Therapeutic Goods (Medical Devices) Regulations.

^h^MDCG_2019-16: Medical Device Coordination Group Document 2019-16 – Guidance on Cybersecurity for medical devices.

^i^FDA_BRA: Factors to Consider When Making Benefit-Risk Determinations in Medical Device Premarket Approval and De Novo Classifications.

^j^FDA_BRA_Uncertainty: Consideration of Uncertainty in Making Benefit-Risk Determinations in Medical Device Premarket Approvals, De Novo Classifications, and Humanitarian Device Exemptions.

^k^FDA_BRA_AC&ED: Factors to Consider Regarding Benefit-Risk in Medical Device Product Availability, Compliance, and Enforcement Decisions.

^l^ISO_14971: International Organization for Standardization Standard 14971:2019 Medical devices—Application of risk management to medical devices.

^m^ISO_24971: International Organization for Standardization Technical Report 24971:2020 Medical devices — Guidance on the application of ISO 14971.

^n^IEC_82304-1: International Electrotechnical Commission Standard 82304-1:2016 Health software Part 1: General requirements for product safety.

^o^AAMI_TIR57: Association for the Advancement of Medical Instrumentation Technical Report TIR57:2016/(R)2023; Principles for medical device security—Risk management.

^p^TGA_Cyber_User: Medical device cybersecurity information for users.

^q^IMDRF_Cyber: Principles and Practices for Medical Device Cybersecurity.

^r^FDA_Cyber_Pre: Cybersecurity in Medical Devices: Quality System Considerations and Content of Premarket Submissions.

^s^FDA_Cyber_Post: Postmarket Management of Cybersecurity in Medical Devices.

^t^HC_Cyber: Pre-market Requirements for Medical Device Cybersecurity.

^u^HSA_GN-20: GN-20: Guidance on Clinical Evaluation.

^v^cMD: connected medical device.

^w^EOS: end of service.

^x^IMDRF_LegMD: Principles and Practices for the Cybersecurity of Legacy Medical Devices.

^y^ISO: International Organization for Standardization.

^z^EU_MDR: European Union Medical Device Regulation.

## Discussion

### Principal Findings

The goal of this scoping review was to identify the current legal frameworks, guidelines, and standards in the United States and EU on how cybersecurity should be considered in the BRA of MDs. Among the 34 documents, we identified 4 (12%) regulations (the EU_MDR, AU_MDR, Canadian Medical Device Regulations, and FD&C Act); 5 (15%) standards issued by the ISO, IEC, and AAMI; 6 (18%) technical documents; and 19 (56%) guidelines issued by the FDA, MDCG, Health Canada, HSA, NIST, MHRA, MFDS, and IMDRF.

The regulations in the United States, Australia, Canada, and the EU provide high-level requirements for the BRA but do not explicitly address the intersection of cybersecurity and BRA [18-20,23]. At the guideline level, MDCG 2019-16 provides an overview of cybersecurity requirements for the EU, acknowledges the relevance of cybersecurity considerations in the BRA, and loosely defines how cybersecurity risks should influence the BRA [13]. Other guidelines (12/34, 35%) underscore the relevance of the BRA without detailing methods for the consideration of cybersecurity risks [21,22,32,37-40,42,43,47,48,59].

Multiple standards and technical reports describing the intersection of cybersecurity and the BRA (3/34, 9%) mandate the execution of a BRA only when residual risks, including cybersecurity-related ones, cannot be reduced to an acceptable level through risk management [11,50,57]. Some of these documents (3/34, 9%) provide methods and examples explicitly for a security BRA [50,52,57], whereas others (2/34, 6%) only loosely describe methods that could be adapted to consider cybersecurity risks without providing more details [11,12].

### BRA and Cybersecurity

In our scoping review, we identified a notable gap between the acknowledgment of the relevance of cybersecurity risks to the BRA and the actual guidance or methods on how to consider these risks within it. Thus, and through the lack of concrete examples, there remains some amount of uncertainty within those guidelines on how and when to conduct a BRA in general and how to conduct a security BRA.

Many of the included documents (9/34, 26%) consider BRA an explicit requirement, necessitating the description and coverage of all safety risks, including those caused by cybersecurity threats [11-13,18,19,21,37,40,46]. Some of the included documents (8/34, 24%) provide sophisticated overviews of cybersecurity risks and the security risk management process, but they do not go into detail regarding the BRA [35,36,44,49,51,59] or refer to generic methods [50,53]. Others (5/34, 15%) provide methods that could be adapted to consider cybersecurity while not containing specific information about the relationship between cybersecurity risks and the BRA [11,12,37,39,40].

Of the 34 documents, 3 (9%) guidance documents separate security and safety risk management into 2 processes [13,52,57]. While the intersection of both processes is acknowledged, they include separate BRAs: one for safety risk management, which considers all residual risks, including security-related ones, comparing them to the clinical benefits to patients and to the health system, and another one for security alone [13,52,57], where manufacturers should appropriately balance the residual security risk against the benefit gained by the design capabilities or security controls of the device [57]. While a lot of guidance exists for the identification of security risks, it remains unclear how security benefits are defined and how they influence the security BRA as well as the safety BRA. The SW96:2023 standard provides a brief hypothetical example, where the benefit of accurate patient identification outweighs the residual risk of storing sensitive patient data on a device [52]. However, this approach somewhat contradicts the understanding of a BRA described in other documents, which mandate a more holistic approach weighing the clinical benefits of the device against the residual cybersecurity risks and conducting an additional general BRA that considers all residual risks [11,12,37,39,40,50].

Some standards and official guidance documents (10/34, 29%) differ in their description of when a BRA must be carried out. One of them only calls for a BRA if residual risks remain unacceptable [51], which contradicts the requirements of the MDCG, TGA, and FDA, which see the BRA as a final step in deciding whether an MD should be put on the market [13,18,19,21,37,39-41,46]. A middle-ground position is provided by multiple documents (5/34, 15%) that require a separate BRA for any individual residual risk that is not judged as acceptable, whereas a BRA should be conducted to evaluate the overall residual risk, which should consider cybersecurity-related residual risks [11,12,50,52,57].

### BRA as Part of the PMS

Traditionally, BRA is often seen as primarily a premarket activity in the MD development and approval process. However, multiple guidelines (6/34, 18%) clarify that BRA is an ongoing process [13,22,32,37,40,48], and especially when initial data are limited, PMS should be used to further define the risk profile of a device and update the BRA accordingly [37]. This is particularly important for cybersecurity risks as these are constantly changing, for example, when previously unknown vulnerabilities in software or communication protocols are discovered and exploited by malicious actors. Therefore, cybersecurity risk monitoring and management is seen as an integral part of PMS by many authorities [13,21,32]. Maintaining an SBOM is seen as supportive for this effort [21,47]. On the basis of newly identified risks, and guided by the BRA as part of risk management, mitigation actions (eg, through patching or device recalls) could be necessary [13,32,42]. In addition, a BRA should be conducted by the health care provider and the MD manufacturer before deploying updates and after the end of service of devices [22,42,48].

### Implementation Challenges

In the included documents, multiple issues were identified that bring to light particular technical challenges to the process of conducting a BRA. First, cybersecurity risks could be difficult to assess, especially in a quantitative manner, due to the unpredictable nature of vulnerabilities [11,22,32,51]. Those vulnerabilities appear over time, for example, through proactive attacks targeting specific aspects of devices or, more commonly, through bugs in standard software libraries or components [61] and zero-day exploits (unknown vulnerabilities that can be exploited before they are publicly known and mitigated [62]). The ISO Technical Report 24971:2020 standard acknowledges the existence of such hard-to-quantify risks, which could also be connected to other new technologies such as artificial intelligence (AI) [63-65], gamification [66], or virtual reality [67,68], but considers them still as relevant for the BRA [12]. Second, updating devices could also lead to harm, primarily regarding the availability of products during the update phase, which needs to be considered in the BRA [22,32,37,42]. Third, there is the need to balance security measures as both too weak and overly restrictive security measures could pose risks [13]. Weak measures do not provide adequate protection (eg, for personal data), whereas overly restrictive measures hinder device usability. Fourth, implementing a risk control measure to reduce one risk can introduce new risks, for example, when adding a higher authentication standard (such as 2-factor authentication) to address the risk of the disclosure of patient data could limit the accessibility of the device in case of an emergency [12]. Finally, for cMDs, the entire IT infrastructure needs to be considered when assessing cybersecurity risks [50,52] (ie, with a network-level risk assessment) as cMDs usually exchange data with multiple other devices and services, some of which are MDs whereas some are not. In addition, the implementation of a new device into an existing infrastructure might introduce vulnerabilities for the entire system.

### Future Research

Innovative approaches for the assessment and management of cybersecurity risks could help overcome these challenges and improve the understanding of the evolving cybersecurity risk landscape of MDs. This includes the use of risk management ontologies to help developers identify existing vulnerabilities in the risk assessment phase [69] and the use of AI-based intrusion detection systems for postmarket risk management, which use machine and deep learning approaches to detect suspicious network behavior, untypical user patterns, malware, and other malicious activities [70,71]. The pre- and postmarket risk assessment could be supported by automatic systems linked to vulnerability databases, which continuously assess a given infrastructure, alert in case of newly defined risks, and propose mitigation measures [72], and AI-based systems that assess and predict vulnerabilities [73,74]. Another approach relevant to cMDs is device monitoring, a standard in industries other than health care, which could help provide an overview of the overall attack surface and vulnerable devices [75]. While some of these approaches are already used in non–health care environments (eg, AI-based intrusion detection systems or device management), others are still in early development phases (eg, automatic risk management and mitigation systems). However, these innovative approaches often do not cover all aspects relevant to the BRA. Thus, further development of methods for the assessment of cybersecurity risks and the incorporation of these risks into the BRA process is necessary. While current methods were often not developed with cybersecurity risks in mind, some, especially qualitative frameworks such as the FDA’s BRF, might be capable of considering cybersecurity risks as they are more flexible [25]. A quantitative framework frequently used for the BRA, the MCDA, is also used for cybersecurity decision-making and evaluation in health care [76,77]. In particular, the MCDA method “Technique for Order Preference by Similarity to Ideal Solution” could be adopted for cybersecurity considerations in the BRA, for example, to define best practices with a favorable benefit-risk profile for authentication, device monitoring, or network architecture, which could take a broad set of criteria, including patient factors, into account. In addition, models such as the Gordon-Loeb model are currently used for the economic analysis of benefits and costs of cybersecurity risk mitigations [78,79]. Future research should explore the feasibility of adapting these models to weigh cybersecurity risks against MD benefits, although this is beyond the scope of this review. The absence of standardized frameworks for BRA in general and for the evaluation of cybersecurity risks likely contributes to the current discrepancies among existing standards, technical documents, and guidance on these themes.

In addition, the interaction among clinical benefits, cybersecurity risks, safety, and security should be explored further. The limitations of current guidance are demonstrated by the following example. A “traditional” predigital laser device for surgical interventions in the eye without any software running on the device and without any interfaces poses only safety risks. However, when adding a digital component (eg, for a cloud connection), new security and safety risks, partly related to cybersecurity, arise. At this time, it remains unclear how those new risks would impact the security BRA as well as the overall BRA.

Understanding how these elements influence each other will help develop more effective risk management strategies and a better understanding of what risks are tolerable in innovative MDs. As the technology develops, new attack vectors will emerge (eg, through quantum computing, AI, or previously undetected dormant faults). Research is needed to identify these potential threats and develop innovative mitigation strategies to counter and proactively approach them. This research should not focus on single devices but also consider entire infrastructures as MDs and non-MDs often exist in the same system.

### Limitations

The results of this scoping review could be limited by several factors. First, our review focused exclusively on IMDRF member states that provide guidance in English (the EU, the United States, Canada, Australia, South Korea, Singapore, and the United Kingdom). This geographic limitation may reduce the applicability of our findings to other regions. Second, the analysis in this scoping review was limited to guidance documents and standards that were relevant to MDs. This could have led to relevant principles described in standards from other areas not being considered. This has no limitation on the validity of the review of the current state of the art for MD cybersecurity risk assessment and BRA, but it could have limited the ability to suggest future directions for the further developments of standards.

### Conclusions

Today, cybersecurity vulnerabilities in cMDs pose a growing risk to patients and health care providers. Therefore, the consideration of these risks in the BRA is essential to decide whether an MD is safe and secure and should be made available on the market. While the recognition of cybersecurity risks in cMDs has increased, there is a substantial gap between this acknowledgment and practical actions. Current regulations, guidelines, and standards mandate the consideration of cybersecurity risks but lack detailed methods for incorporating them into the BRA. To bridge this gap, it is essential for manufacturers and regulators to develop standardized frameworks that provide clear guidance on evaluating the impact of cybersecurity risks on the device’s safety and security, ultimately enhancing patient safety and device effectiveness.

## Appendix

Multimedia Appendix 1 PRISMA-ScR (Preferred Reporting Items for Systematic Reviews and Meta-Analyses extension for Scoping Reviews) checklist and a detailed table with URLs for the searched organizations and search results.

## Abbreviations

AAMI: Association for the Advancement of Medical Instrumentation
AI: artificial intelligence
AU_MDR: Australian Therapeutic Goods (Medical Devices) Regulations
BRA: benefit-risk analysis
BRF: Benefit-Risk Framework
cMD: connected medical device
EU: European Union
EU_MDR: European Union Medical Device Regulation
FD&C Act: Federal Food, Drug, and Cosmetic Act
FDA: Food and Drug Administration
HSA: Health Sciences Authority
IEC: International Electrotechnical Commission
IMDRF: International Medical Device Regulators Forum
ISO: International Organization for Standardization
MCDA: multicriteria decision analysis
MD: medical device
MDCG: Medical Device Coordination Group
MFDS: Ministry of Food and Drug Safety
MHRA: Medicines and Healthcare Products Regulatory Agency
NIST: National Institute of Standards and Technology
PMS: postmarket surveillance
PRISMA-ScR: Preferred Reporting Items for Systematic Reviews and Meta-Analyses extension for Scoping Reviews
SBOM: software bill of materials
TGA: Therapeutic Goods Administration

## References

[1] Machal ML (2023). An overview about connected medical devices and their risks. Stud Health Technol Inform.

[2] Huang C, Wang J, Wang S, Zhang Y (2023). Internet of medical things: a systematic review. Neurocomputing.

[3] Haghi Kashani M, Madanipour M, Nikravan M, Asghari P, Mahdipour E (2021). A systematic review of IoT in healthcare: applications, techniques, and trends. J Netw Comput Appl.

[4] Dwivedi R, Mehrotra D, Chandra S (2022). Potential of Internet of Medical Things (IoMT) applications in building a smart healthcare system: a systematic review. J Oral Biol Craniofac Res.

[5] Perakslis E, Ginsburg GS (2021). Digital health-the need to assess benefits, risks, and value. JAMA.

[6] Bauwens J, Ruckebusch P, Giannoulis S, Moerman I, Poorter ED (2020). Over-the-air software updates in the internet of things: an overview of key principles. IEEE Commun Mag.

[7] Bracciale L, Loreti P, Bianchi G (2023). Cybersecurity vulnerability analysis of medical devices purchased by national health services. Sci Rep.

[8] He Y, Aliyu A, Evans M, Luo C (2021). Health care cybersecurity challenges and solutions under the climate of COVID-19: scoping review. J Med Internet Res.

[9] Munoz Cornejo G, Lee J, Russell BA (2024). A thematic analysis of ransomware incidents among United States hospitals, 2016–2022. Health Technol.

[10] Ralston W (2020). The untold story of a cyberattack, a hospital and a dying woman. Wired.

[11] (2019). ISO 14971:2019 medical devices — application of risk management to medical devices. International Organization for Standardization.

[12] (2020). ISO/TR 24971:2020 medical devices — guidance on the application of ISO 14971. International Organization for Standardization.

[13] (2020). MDCG 2019-16 rev. 1 - Guidance on cybersecurity for medical devices. European Commission.

[14] Neprash HT, McGlave CC, Cross DA, Virnig BA, Puskarich MA, Huling JD, Rozenshtein AZ, Nikpay SS (2022). Trends in ransomware attacks on US hospitals, clinics, and other health care delivery organizations, 2016-2021. JAMA Health Forum.

[15] Manufacturer and user facility device experience (MAUDE) database. U.S. Food & Drug Administration.

[16] Klonoff D, Han J (2019). The first recall of a diabetes device because of cybersecurity risks. J Diabetes Sci Technol.

[17] Medical device recalls. U.S. Food & Drug Administration.

[18] Regulation (EU) 2017/745 of the European Parliament and of the Council of 5 April 2017 on medical devices, amending directive 2001/83/EC, Regulation (EC) No 178/2002 and Regulation (EC) No 1223/2009 and repealing Council Directives 90/385/EEC and 93/42/EEC (text with EEA relevance). European Union.

[19] 21 U.S. Code chapter 9 - Federal Food, Drug, and Cosmetic Act. Legal Informative Institute.

[20] Therapeutic goods (medical devices) regulations 2002. Australian Government.

[21] (2022). Medical device cyber security guidance for industry. Australian Government, Department of Health and Aged Care.

[22] (2023). Cybersecurity in medical devices: quality system considerations and content of premarket submissions. U.S. Food & Drug Administration.

[23] Medical devices regulations (SOR/98-282). Government of Canada.

[24] Su G, Deng D (2023). Regulatory requirements and optimization of multiple criteria decision analysis to quantify the benefit-risk assessment of medical devices. Expert Rev Med Devices.

[25] Freyer O, Jahed F, Ostermann M, Feig M, Gilbert S Methodologies for the benefit-risk analysis of medical devices: a systematic review. Research Square.

[26] Mt-Isa S, Ouwens M, Robert V, Gebel M, Schacht A, Hirsch I (2016). Structured benefit-risk assessment: a review of key publications and initiatives on frameworks and methodologies. Pharm Stat.

[27] Agapova M, Devine EB, Bresnahan BW, Higashi MK, Garrison LP Jr (2014). Applying quantitative benefit-risk analysis to aid regulatory decision making in diagnostic imaging: methods, challenges, and opportunities. Acad Radiol.

[28] Fu B, Li X, Scott J, He W (2020). A new framework to address challenges in quantitative benefit-risk assessment for medical products. Contemp Clin Trials.

[29] Gebel M, Renz C, Rodriguez L, Simonetti A, Yang H, Edwards B, Higginson JM, Charpentier N, Colopy M (2024). A survey to assess the current status of structured benefit-risk assessment in the global drug and medical device industry. Ther Innov Regul Sci.

[30] Sun S, Heske S, Mercadel M, Wimmer J (2021). Predicting regulatory product approvals using a proposed quantitative version of FDA's benefit-risk framework to calculate net-benefit score and benefit-risk ratio. Ther Innov Regul Sci.

[31] Tervonen T, Veldwijk J, Payne K, Ng X, Levitan B, Lackey LG, Marsh K, Thokala P, Pignatti F, Donnelly A, Ho M (2023). Quantitative benefit-risk assessment in medical product decision making: a good practices report of an ISPOR task force. Value Health.

[32] (2016). Postmarket management of cybersecurity in medical devices. U.S. Food & Drug Administration.

[33] Tricco AC, Lillie E, Zarin W, O'Brien KK, Colquhoun H, Levac D, Moher D, Peters MD, Horsley T, Weeks L, Hempel S, Akl EA, Chang C, McGowan J, Stewart L, Hartling L, Aldcroft A, Wilson MG, Garritty C, Lewin S, Godfrey CM, Macdonald MT, Langlois EV, Soares-Weiser K, Moriarty J, Clifford T, Tunçalp Ö, Straus SE (2018). PRISMA extension for scoping reviews (PRISMA-ScR): checklist and explanation. Ann Intern Med.

[34] Page MJ, McKenzie JE, Bossuyt PM, Boutron I, Hoffmann TC, Mulrow CD, Shamseer L, Tetzlaff JM, Akl EA, Brennan SE, Chou R, Glanville J, Grimshaw JM, Hróbjartsson A, Lalu MM, Li T, Loder EW, Mayo-Wilson E, McDonald S, McGuinness LA, Stewart LA, Thomas J, Tricco AC, Welch VA, Whiting P, Moher D (2021). The PRISMA 2020 statement: an updated guideline for reporting systematic reviews. BMJ.

[35] NIST SP 800-30. National Institute of Standards and Technology.

[36] (2023). The NIST Cybersecurity Framework (CSF) 2.0. National Institute of Standards and Technology.

[37] (2016). Factors to consider regarding benefit risk in medical device product availability, compliance, and enforcement decisions. U.S. Food & Drug Administration.

[38] (2022). GN-20: guidance on clinical evaluation. Health Science Authority.

[39] (2019). Consideration of uncertainty in making benefit-risk determinations in medical device premarket approvals, de novo classifications, and humanitarian device exemptions. U.S. Food & Drug Administration.

[40] (2019). Factors to consider when making benefit-risk determinations in medical device premarket approval and de novo classifications. U.S. Food & Drug Administration.

[41] (2022). Medical device cyber security information for users. Commonwealth of Australia.

[42] (2020). Principles and practices for medical device cybersecurity. International Medical Device Regulators Forum.

[43] Guidance documents for medical devices. Health Sciences Authority.

[44] Guideline on review and approval for cybersecurity of medical devices (for industry). Ministry of Food and Drug Safety.

[45] (2023). Software and AI as a medical device change programme - roadmap. Medicines & Healthcare products Regulatory Agency, United Kingdom Government.

[46] (2022). Complying with the Essential Principles on the safety and performance of medical devices. The Department of Health and Aged Care, Australian Government.

[47] (2023). Principles and practices for software bill of materials for medical device cybersecurity. International Medical Device Regulators Forum.

[48] (2023). Principles and practices for the cybersecurity of legacy medical devices. International Medical Device Regulators Forum.

[49] (2016). IEC 82304-1:2016 Health software part 1: general requirements for product safety. International Electrotechnical Commission.

[50] (2021). IEC 80001-1:2021: application of risk management for IT-networks incorporating medical devices - part 1: Safety, effectiveness and security in the implementation and use of connected medical devices or connected health software. International Electrotechnical Commission.

[51] (2021). IEC 81001-5-1:2021: health software and health IT systems safety, effectiveness and security. International Electrotechnical Commission.

[52] (2022). ANSI/AAMI SW96:2023; standard for medical device security—security risk management for device manufacturers. Association for the Advancement of Medical Instrumentation.

[53] (2009). IEC/TR 80002-1:2009: medical device software part 1: guidance on the application of ISO 14971 to medical device software. International Electrotechnical Commission.

[54] (2012). IEC/TR 80001-2-2:2012: application of risk management for IT-networks incorporating medical devices: part 2-2: guidance for the communication of medical device security needs, risks and controls. International Electrotechnical Commission.

[55] (2012). IEC/TR 80001-2-1:2012: application of risk management for IT-networks incorporating medical devices: part 2-1: step by step risk management of medical IT-networks; practical applications and examples. International Electrotechnical Commission.

[56] (2015). ISO/TR 80001-2-7:2015: application of risk management for IT-networks incorporating medical devices — application guidance. International Organization for Standardization.

[57] (2023). AAMI TIR57:2016 (R2023) principles for medical device security - risk management. Association for the Advancement of Medical Instrumentation (AAMI).

[58] (2017). Regulation (EU) 2017/746 of the European Parliament and of the Council of 5 April 2017 on in vitro diagnostic medical devices and repealing Directive 98/79/EC and Commission Decision 2010/227/EU (Text with EEA relevance. ). European Parliament, European Council.

[59] (2019). Guidance document: pre-market requirements for medical device cybersecurity. Government of Canada.

[60] (2022). DIN EN ISO 14971:2022-04. Deutsches Institut für Normung.

[61] Lins M, Mayrhofer R, Roland M, Hofer D, Schwaighofer M On the critical path to implant backdoors and the effectiveness of potential mitigation techniques: early learnings from XZ. arXiv.

[62] Bilge L, Dumitraş T (2012). Before we knew it: an empirical study of zero-day attacks in the real world. Proceedings of the 2012 ACM Conference on Computer and Communications Security.

[63] Meskó B, Topol EJ (2023). The imperative for regulatory oversight of large language models (or generative AI) in healthcare. NPJ Digit Med.

[64] Gilbert S, Harvey H, Melvin T, Vollebregt E, Wicks P (2023). Large language model AI chatbots require approval as medical devices. Nat Med.

[65] Freyer O, Wiest IC, Kather JN, Gilbert S (2024). A future role for health applications of large language models depends on regulators enforcing safety standards. The Lancet Digit Health.

[66] Freyer O, Wrona KJ, de Snoeck Q, Hofmann M, Melvin T, Stratton-Powell A, Wicks P, Parks AC, Gilbert S (2024). The regulatory status of health apps that employ gamification. Sci Rep.

[67] Freyer O, Gilbert S (2023). Bridging between hype and implementation in medical extended reality. NPJ Digit Med.

[68] Souchet AD, Lourdeaux D, Pagani A, Rebenitsch L (2022). A narrative review of immersive virtual reality’s ergonomics and risks at the workplace: cybersickness, visual fatigue, muscular fatigue, acute stress, and mental overload. Virtual Reality.

[69] Bughio KS, Cook DM, Shah SA (2024). Developing a novel ontology for cybersecurity in internet of medical things-enabled remote patient monitoring. Sensors (Basel).

[70] Sowmya T, Mary Anita EA (2023). A comprehensive review of AI based intrusion detection system. Meas Sens.

[71] Salem AH, Azzam SM, Emam OE, Abohany AA (2024). Advancing cybersecurity: a comprehensive review of AI-driven detection techniques. J Big Data.

[72] Ksibi S, Jaidi F, Bouhoula A (2022). A comprehensive study of security and cyber-security risk management within e-health systems: synthesis, analysis and a novel quantified approach. Mobile Netw Appl.

[73] Abdulsatar M, Ahmad H, Goel D, Ullah F Towards deep learning enabled cybersecurity risk assessment for microservice architectures. arXiv.

[74] Sarker IH, Furhad MH, Nowrozy R (2021). AI-driven cybersecurity: an overview, security intelligence modeling and research directions. SN Comput Sci.

[75] (2023). Best IoT device management platforms: a comprehensive comparison. Novotech.

[76] Alzahrani FA, Ahmad M, Ansari MT (2022). Towards design and development of security assessment framework for internet of medical things. Appl Sci.

[77] Wang L, Ali Y, Nazir S, Niazi M (2020). ISA evaluation framework for security of internet of health things system using AHP-TOPSIS methods. IEEE Access.

[78] Gordon LA, Loeb MP, Zhou L (2020). Integrating cost–benefit analysis into the NIST Cybersecurity Framework via the Gordon–Loeb Model. J Cybersecurity.

[79] Krutilla K, Alexeev A, Jardine E, Good D (2021). The benefits and costs of cybersecurity risk reduction: a dynamic extension of the Gordon and Loeb Model. Risk Anal.

