# Supplementary material for: Consideration of Cybersecurity Risks in the Benefit-Risk Analysis of Medical Devices: Scoping Review
Source: J Med Internet Res. 2024 Dec 24;26:e65528. doi: 10.2196/65528 (PMC11707448; doi:10.2196/65528)
Supplement: Multimedia Appendix 1 [file jmir_v26i1e65528_app1.pdf]

Consideration of Cybersecurity in the Benefit-Risk Analysis of Medical Devices: A Scoping Review - Multimedia Appendix 1

Authors: Oscar Freyer[1¶], Fatemeh Jahed[1], Max Ostermann[1], Christian Rosenzweig[2], Pascal Werner[3], Stephen Gilbert[1]

[1]- Else Kröner Fresenius Center for Digital Health, TUD Dresden University of Technology, Dresden, Germany
[2]- Johner Institute, Konstanz, Germany
[3]- Regulatory.me, Mebane, North Carolina, USA
[¶]- corresponding author: Oscar Freyer, oscar.freyer@tu-dresden.de, TUD Dresden University of Technology, Else Kröner Fresenius Center for Digital Health, Fetscherstr. 74, 01307 Dresden

Content

Table S1. PRISMA-ScR Checklist pp. 2 - 3

Table S2. Search Results with Links pp. 4 - 6

Table S1. PRISMA-ScR Checklist

Preferred Reporting Items for Systematic reviews and Meta-Analyses extension for Scoping Reviews (PRISMA-ScR) Checklist

SECTION	ITEM	PRISMA-ScR CHECKLIST ITEM	REPORTED ON PAGE #
**TITLE**			
Title	1	Identify the report as a scoping review.	1
**ABSTRACT**			
Structured summary	2	Provide a structured summary that includes (as applicable): background, objectives, eligibility criteria, sources of evidence, charting methods, results, and conclusions that relate to the review questions and objectives.	1
**INTRODUCTION**			
Rationale	3	Describe the rationale for the review in the context of what is already known. Explain why the review questions/objectives lend themselves to a scoping review approach.	3
Objectives	4	Provide an explicit statement of the questions and objectives being addressed with reference to their key elements (e.g., population or participants, concepts, and context) or other relevant key elements used to conceptualize the review questions and/or objectives.	3
**METHODS**			
Protocol and registration	5	Indicate whether a review protocol exists; state if and where it can be accessed (e.g., a Web address); and if available, provide registration information, including the registration number.	n.a.
Eligibility criteria	6	Specify characteristics of the sources of evidence used as eligibility criteria (e.g., years considered, language, and publication status), and provide a rationale.	4
Information sources*	7	Describe all information sources in the search (e.g., databases with dates of coverage and contact with authors to identify additional sources), as well as the date the most recent search was executed.	3
Search	8	Present the full electronic search strategy for at least 1 database, including any limits used, such that it could be repeated.	3
Selection of sources of evidence†	9	State the process for selecting sources of evidence (i.e., screening and eligibility) included in the scoping review.	4
Data charting process‡	10	Describe the methods of charting data from the included sources of evidence (e.g., calibrated forms or forms that have been tested by the team before their use, and whether data charting was done independently or in duplicate) and any processes for obtaining and confirming data from investigators.	n.a.
Data items	11	List and define all variables for which data were sought and any assumptions and simplifications made.	4
Critical appraisal of individual sources of evidence§	12	If done, provide a rationale for conducting a critical appraisal of included sources of evidence; describe the methods used and how this information was used in any data synthesis (if appropriate).	n.a.
Synthesis of results	13	Describe the methods of handling and summarizing the data that were charted.	4

SECTION	ITEM	PRISMA-ScR CHECKLIST ITEM	REPORTED ON PAGE #
**RESULTS**			
Selection of sources of evidence	14	Give numbers of sources of evidence screened, assessed for eligibility, and included in the review, with reasons for exclusions at each stage, ideally using a flow diagram.	4-5
Characteristics of sources of evidence	15	For each source of evidence, present characteristics for which data were charted and provide the citations.	5-9
Critical appraisal within sources of evidence	16	If done, present data on critical appraisal of included sources of evidence (see item 12).	n.a.
Results of individual sources of evidence	17	For each included source of evidence, present the relevant data that were charted that relate to the review questions and objectives.	5-9
Synthesis of results	18	Summarize and/or present the charting results as they relate to the review questions and objectives.	9-17
**DISCUSSION**			
Summary of evidence	19	Summarize the main results (including an overview of concepts, themes, and types of evidence available), link to the review questions and objectives, and consider the relevance to key groups.	17, 18
Limitations	20	Discuss the limitations of the scoping review process.	21
Conclusions	21	Provide a general interpretation of the results with respect to the review questions and objectives, as well as potential implications and/or next steps.	18-21
**FUNDING**			
Funding	22	Describe sources of funding for the included sources of evidence, as well as sources of funding for the scoping review. Describe the role of the funders of the scoping review.	21-22

JBI = Joanna Briggs Institute; PRISMA-ScR = Preferred Reporting Items for Systematic reviews and Meta-Analyses extension for Scoping Reviews.
* Where *sources of evidence* (see second footnote) are compiled from, such as bibliographic databases, social media platforms, and Web sites.
† A more inclusive/heterogeneous term used to account for the different types of evidence or data sources (e.g., quantitative and/or qualitative research, expert opinion, and policy documents) that may be eligible in a scoping review as opposed to only studies. This is not to be confused with *information sources* (see first footnote).
‡ The frameworks by Arksey and O'Malley (6) and Levac and colleagues (7) and the JBI guidance (4, 5) refer to the process of data extraction in a scoping review as data charting.
§ The process of systematically examining research evidence to assess its validity, results, and relevance before using it to inform a decision. This term is used for items 12 and 19 instead of "risk of bias" (which is more applicable to systematic reviews of interventions) to include and acknowledge the various sources of evidence that may be used in a scoping review (e.g., quantitative and/or qualitative research, expert opinion, and policy document).

From: Tricco AC, Lillie E, Zarin W, O'Brien KK, Colquhoun H, Levac D, et al. PRISMA Extension for Scoping Reviews (PRISMAScR): Checklist and Explanation. Ann Intern Med. 2018;169:467–473. doi: 10.7326/M18-0850.

Table S2. Search Results with Links

Name	Abbreviation used in article	Type	URL
U.S. Food and Drug Administration	FDA	Organisation	https://www.fda.gov
National Institute of Standards and Technology (US)	NIST	Organisation	https://www.nist.gov
Health Sciences Authority (Singapore)	HSA	Organisation	https://www.hsa.gov.sg
Medical Device Coordination Group (EU)	MDCG	Organisation	https://health.ec.europa.eu/medical-devices-sector/new-regulations/guidance-mdcg-endorsed-documents-and-other-guidance_en
Health Canada	Health Canada	Organisation	https://www.canada.ca/en/health-canada.html
Therapeutic Goods Administration (Australia)	TGA	Organisation	https://www.tga.gov.au
International Medical Device Regulators Forum	IMDRF	Organisation	https://www.imdrf.org
Medicines & Healthcare products Regulatory Agency (UK)	MHRA	Organisation	https://www.gov.uk/government/organisations/medicines-and-healthcare-products-regulatory-agency
Ministry of Food and Drug Safety (Republic of Korea)	MFDS	Organisation	https://www.mfds.go.kr/eng/index.do
International Electrotechnical Commission	IEC	Organisation	https://iec.ch/homepage
International Organization for Standardization	ISO	Organisation	https://www.iso.org/home.html
Association for the Advancement of Medical Instrumentation	AAMI	Organisation	https://www.aami.org
Federal Food, Drug, and Cosmetic Act	US_FD&C	Regulation	https://www.law.cornell.edu/uscode/text/21
Medical Devices Regulations (SOR/98-282)	CA_MDR	Regulation	https://laws-lois.justice.gc.ca/eng/regulations/sor-98-282/
Therapeutic Goods (Medical Devices) Regulations 2002	AU_MDR	Regulation	https://www.legislation.gov.au/F2002B00237/latest
Regulation (EU) 2017/745 of the European Parliament and of the Council of 5 April 2017 on medical devices, amending Directive 2001/83/EC, Regulation (EC) No 178/2002 and Regulation (EC) No 1223/2009 and repealing Council Directives 90/385/EEC and 93/42/EEC (Text with EEA relevance.)	EU_MDR	Regulation	https://eur-lex.europa.eu/legal-content/EN/TXT/?uri=celex%3A32017R0745
NIST SP 800 - 30	NIST_800-30	Guidance	https://www.nist.gov/privacy-framework/nist-sp-800-30
NIST Cyber security framework	NIST_CSF2.0	Guidance	https://www.nist.gov/cyberframework
Factors to Consider Regarding BenefitRisk in Medical Device Product Availability, Compliance, and Enforcement Decisions	FDA_BRA_AC&ED	Guidance	https://www.fda.gov/regulatory-information/search-fda-guidance-documents/factors-consider-regarding-benefit-risk-medical-device-product-availability-compliance-and

Postmarket Management of Cybersecurity in Medical Devices	FDA_Cyber_Post	Guidance	https://www.fda.gov/regulatory-information/search-fda-guidance-documents/postmarket-management-cybersecurity-medical-devices
GN-20: Guidance on Clinical Evaluation	HSA_GN-20	Guidance	https://www.hsa.gov.sg/medical-devices/guidance-documents
Consideration of Uncertainty in Making Benefit-Risk Determinations in Medical Device Premarket Approvals, De Novo Classifications, and Humanitarian Device Exemptions	FDA_BRA_Uncertainty	Guidance	https://www.fda.gov/regulatory-information/search-fda-guidance-documents/consideration-uncertainty-making-benefit-risk-determinations-medical-device-premarket-approvals-de
Factors to Consider When Making Benefit-Risk Determinations in Medical Device Premarket Approval and De Novo Classifications	FDA_BRA	Guidance	https://www.fda.gov/regulatory-information/search-fda-guidance-documents/factors-consider-when-making-benefit-risk-determinations-medical-device-premarket-approval-and-de
MDCG 2019-16 Rev.1	MDCG_2019-16	Guidance	https://health.ec.europa.eu/document/download/b23b362f-8a56-434c-922a-5b3ca4d0a7a1_en
Pre-market Requirements for Medical Device Cybersecurity	HC_Cyber	Guidance	https://www.canada.ca/en/health-canada/services/drugs-health-products/medical-devices/application-information/guidance-documents/cybersecurity/document.html
Medical device cyber security guidance for industry	TGA_Cyber_Dev	Guidance	https://www.tga.gov.au/resources/guidance/complying-medical-device-cyber-security-requirements
Medical device cyber security information for users	TGA_Cyber_User	Guidance	https://www.tga.gov.au/resources/resource/reference-material/medical-device-cyber-security-information-users
Principles and practices for medical device cybersecurity	IMDRF_Cyber	Guidance	https://www.imdrf.org/documents/principles-and-practices-medical-device-cybersecurity
Regulatory Guidelines for Software Medical Devices – A Life Cycle Approach	HSA_Cyber	Guidance	https://www.hsa.gov.sg/medical-devices/guidance-documents
Guideline on Review and Approval for Cybersecurity of Medical Devices (For industry)	MFDS_Cyber	Guidance	https://www.mfds.go.kr/eng/brd/m_40/list.do?page=2& srchFr=&srchTo=&srchWord=&srchTp=&itm_seq_1=0&itm_seq_2=0&multi_itm_seq=0&company_cd=&company_nm=
Software and AI as a Medical Device Change Programme	MHRA_SaMD	Guidance	https://www.gov.uk/government/publications/software-and-ai-as-a-medical-device-change-programme/software-and-ai-as-a-medical-device-change-programme-roadmap
Complying with the Essential Principles on the safety and performance of medical devices	TGA_Safety	Guidance	https://www.tga.gov.au/resources/guidance/complying-essential-principles-safety-and-performance-medical-devices
Cybersecurity in medical devices: quality system considerations and content of premarket submissions	FDA_Cyber_Pre	Guidance	https://www.fda.gov/regulatory-information/search-fda-guidance-documents/cybersecurity-medical-devices-quality-system-considerations-and-content-premarket-submissions
Principles and practices for software bill of material for medical device cybersecurity	IMDRF_SBOM	Guidance	https://www.imdrf.org/documents/principles-and-practices-software-bill-materials-medical-device-cybersecurity
Principles and practices for cybersecurity of legacy medical device	IMDRF_LegMD	Guidance	https://www.imdrf.org/documents/principles-and-practices-cybersecurity-legacy-medical-devices
IEC 82304-1:2016	IEC_82304-1	Standard	https://www.iso.org/standard/59543.html
ISO 14971:2019	ISO_14971	Standard	https://www.iso.org/standard/72704.html
IEC 80001-1:2021	IEC_80001-1	Standard	https://webstore.iec.ch/en/publication/34263

IEC 81001-5-1:2021	IEC_81001-5-1	Standard	https://www.iso.org/standard/76097.html
ANSI/AAMI SW96:2023	AAMI_SW96	Standard	https://array.aami.org/doi/10.2345/9781570208621
IEC 80002-1:2009	IEC_80002-1	Technical Report	https://www.iso.org/standard/54146.html
IEC/TR 80001-2-2:2012	IEC_80001-2-2	Technical Report	https://www.iso.org/standard/57939.html
IEC/TR 80001-2-1:2012	IEC_80001-2-1	Technical Report	https://www.iso.org/standard/57934.html
ISO/TR 80001-2-7:2015	ISO_80001-2-7	Technical Report	https://www.iso.org/standard/63509.html
Principles for medical device security - Risk Management; AAMI TIR57:2016/(R)2023	AAMI_TIR57	Technical Report	https://array.aami.org/doi/abs/10.2345/9781570206122.ch1
ISO TR 24971:2020	ISO_24971	Technical Report	https://www.iso.org/standard/74437.html
